# A rare case of bacterial translocation from a renal abscess resulting in empyema necessitans: a case report and literature review

**DOI:** 10.1093/jscr/rjaf908

**Published:** 2025-11-14

**Authors:** Monica Bobila, Walid Akram Hussain, Niyousha Ahmadi, Gregory Crisafulli, Aakash Trivedi, Vinay Tak

**Affiliations:** General Surgery Department, St. Joseph University Medical Center, 703 Main St, Paterson, NJ 07503, United States; General Surgery Department, St. Joseph University Medical Center, 703 Main St, Paterson, NJ 07503, United States; General Surgery Department, St. Joseph University Medical Center, 703 Main St, Paterson, NJ 07503, United States; General Surgery Department, St. Joseph University Medical Center, 703 Main St, Paterson, NJ 07503, United States; General Surgery Department, St. Joseph University Medical Center, 703 Main St, Paterson, NJ 07503, United States; Cardiothoracic Surgery Department, St. Joseph University Medical Center, 703 Main St, Paterson, NJ 07503, United States

**Keywords:** empyema necessitans, *E. coli* empyema, pleurocutaneous fistula, bacterial transmigration, thoracic empyema

## Abstract

Empyema necessitans (EN) is a rare complication of thoracic empyema in which infection spreads into the chest wall. While historically associated with tuberculosis, EN caused by *Escherichia coli* is exceedingly rare. Our report highlights a case of EN in a 76-year-old male that developed after dislodgement of a chest tube placed for the treatment of a pleural effusion associated with a perinephric abscess. The patient presented with a cough and chest wall bulge and admission imaging confirmed a loculated pleural effusion with extension into the chest wall. Cultures from a spontaneously draining pleurocutaneous fistula grew *E. coli* and the patient required surgical intervention for decortication and excision of the fistula tract. This case highlights the importance of considering extra-thoracic sources of infection in empyema, maintaining suspicion for EN in patients with a history of inadequate drainage, and the role of early surgical intervention in preventing complications.

## Introduction

Empyema necessitans (EN) is described as a rare complication of inadequately managed empyema thoracis, in which infection spreads through the soft tissues and skin of the chest [1]. It is most commonly caused by indolent infections such as tuberculosis or actinomycosis, though other causes including staphylococcus, pneumococcus, and rarely *Escherichia coli* and other anaerobic species have been reported. A small subset of patients with empyema can develop EN, if inadequately treated [2]. Although with the advent of antibiotics, the incidence of EN has sharply declined, clinical suspicion should be high in patients presenting with a history of inadequately treated pleural effusion/empyema or those residing in medically underserved areas [3]. Here, we present a unique case of a patient with EN, with cultures growing *E. coli*, that developed as a complication of a dislodged chest tube placed for treatment of a pleural effusion and a concurrent perinephric abscess, which ultimately required surgical decortication and debridement for definitive management.

## Case presentation

A 76-year-old man with a history of coronary artery disease, atrial fibrillation, heart failure, hypertension, and benign prostatic hyperplasia presented to the emergency department with a 3-week history of cough with white sputum production and a bulge superior to a previous chest tube site. One month prior, he was hospitalized for a left renal abscess and left pleural effusion, which were managed with drainage of the abscess and chest tube placement. The chest tube became dislodged during his initial hospitalization and retained fluid was noted on imaging. Due to symptomatic improvement, he was discharged with plans for outpatient pulmonology follow-up.

**Figure 1:**
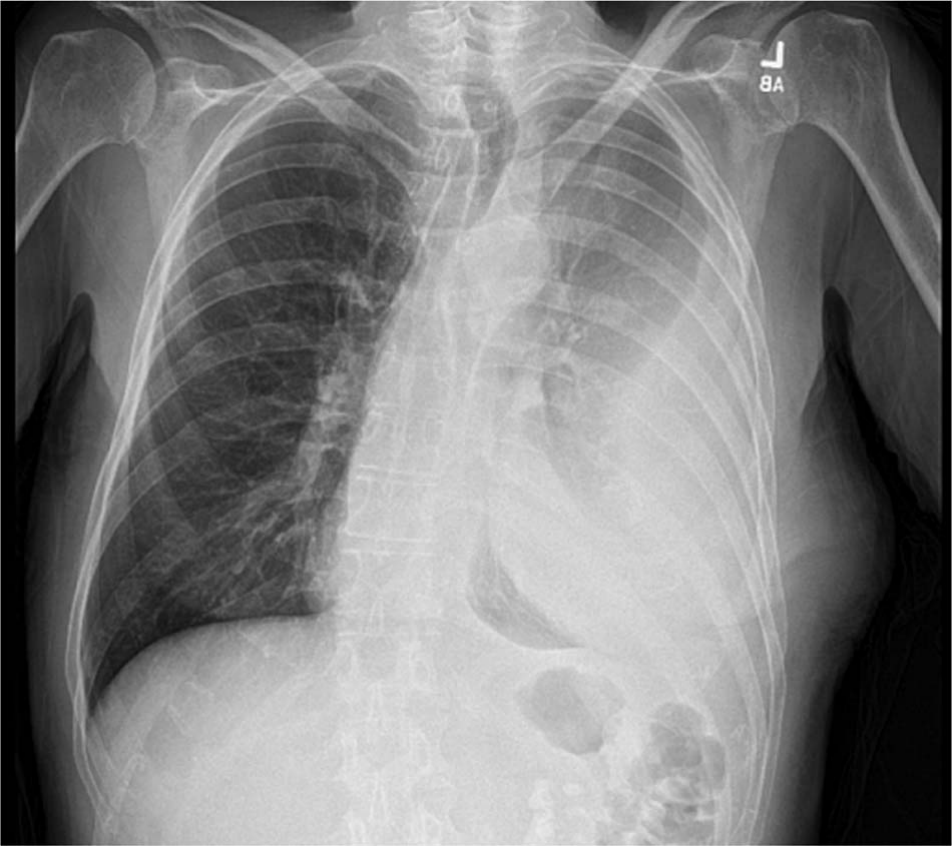
Initial chest radiograph showing a large loculated left sided pleural effusion.

**Figure 2:**
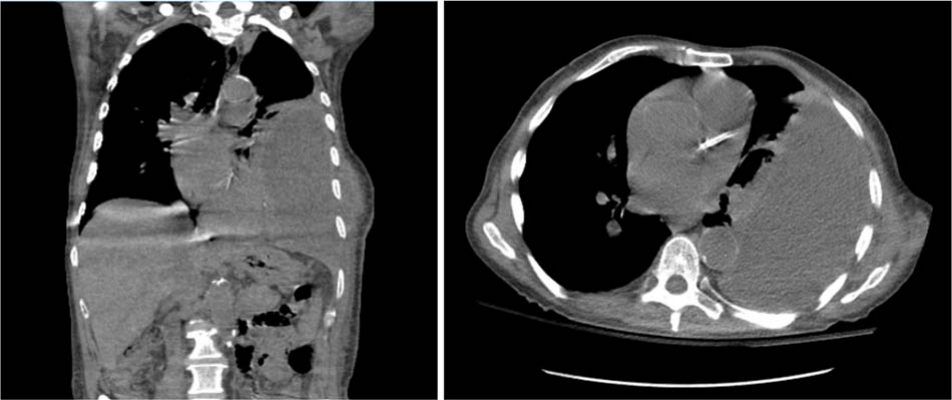
Coronal (left) and axial (right) view CT scan of the chest showing a large complex loculated left pleural effusion with bulging into the chest wall, raising concern for empyema necessitans.

On presentation, chest radiography (Fig. 1) showed a large loculated left pleural effusion, and computed tomography (CT) scan (Fig. 2) confirmed the presence of the effusion with extension into the chest wall. The patient appeared ill with increased work of breathing. Physical exam revealed a large, fluctuant area with a solid component superior to the previous chest tube site, with no overlying skin changes. Blood work was significant for hyponatremia to 127 and a white blood cell count of 10.3 with no neutrophilic predominance. The patient was admitted, started on IV antibiotics, and dual antiplatelet therapy was held in preparation of surgical treatment of his EN. During this time, the fluctuant mass developed an opening, creating a pleurocutaneous fistula, and began to drain significant purulent fluid, which was sent for bacterial cultures.

The patient underwent a left posterolateral thoracotomy with decortication, during which 650 ml of purulent fluid was evacuated and sent for gram stain and culture. He further underwent excision and closure of the fistula tract, and placement of anterior and posterior chest tubes. Intra-operatively, his lung was found to be completely entrapped by a thick pleural peel, which was excised. The patient recovered well and had an uncomplicated postoperative course. Wound cultures obtained prior to surgery from the draining sinus tract were positive for *E. coli*, and the patient was treated with ceftriaxone and metronidazole. The patient showed clinical improvement, decreasing leukocytosis, and stable oxygen saturation, never requiring supplemental oxygen outside of the perioperative period. He was discharged home on post-operative 17 with a multiple-week course of ceftriaxone and metronidazole, with follow-up planned with thoracic surgery and pulmonology, and repeat CT imaging in 6–8 months.

## Discussion

Empyema is a subtype of parapneumonic effusion characterized by a collection of purulent fluid in the pleural space [4]. EN is a rare but serious complication of empyema wherein the infection extends into the chest wall and presents as a subcutaneous swelling, often with fluctuance and surrounding cellulitis [5, 6]. Additional symptoms include pleuritic chest pain, nonproductive cough, and systemic signs, such as tachycardia, tachypnea, and increased work of breathing [7]. Patients with chronic conditions, including heart failure, diabetes, or immunosuppression, are at higher risk of developing empyema necessitans [8].

The most common pathogenic organisms causing EN include *Streptococcus pneumoniae, Staphylococcus aureus, Pseudomonas aeruginosa*, and *mycobacteria* [9]. However, *E. coli* is a rare causative organism, often affecting patients with chronic illnesses or immunosuppression [10]. Our patient’s history of left perinephric abscess requiring interventional radiology (IR) drainage suggests that the infection may have originated from this site and subsequently spread to the pleural space. The isolation of *E. coli* from the culture of the fluid also supports this hypothesis. *E. coli* is also the most likely causative agent in patients with acute pyelonephritis with associated pleural effusion [11, 12]. Our patient did have an extensive history of cardiovascular disease which might have acted as a risk factor for the patient developing empyema as supported by established literature.

EN is a potentially life-threatening condition, and prompt diagnosis and treatment is crucial. Initial imaging with a chest X-ray may reveal a loculated pleural effusion. Thoracic ultrasonography is useful in identifying simple effusions from complex ones, detecting septations and loculations characteristic of empyema, and guiding safe thoracentesis and has been demonstrated to be more sensitive in empyema compared to chest X-Ray [13]. However, a CT scan demonstrating a pleural effusion that is connected to the chest wall mass is necessary for the diagnosis for EN [14]. Additionally, pleural fluid analysis is crucial for confirming the infection and identifying the causative organism to guide targeted antibiotic therapy. In our case, pleural fluid cultures showed no growth, but a culture from the patient’s draining wound revealed *E. coli* sensitive to ceftriaxone. Notably, prior cultures from his left renal abscess grew *E. coli*, supporting the hypothesis of transmigration from the perinephric abscess to the pleural space.

Management of EN requires both antibiotic therapy and drainage of the infected fluid, whether through chest tube placement or surgical drainage. A diagnostic thoracentesis with chest tube drainage is an effective treatment in more than 50% of cases. However, in cases with extensive loculations or lung entrapment, surgical intervention is necessary. This can include video assisted thoracoscopic surgery, Open drainage, decortication, as well as lobectomy and pneumonectomy in rampant, uncontrolled infection [8].

This case highlights an unusual presentation of EN from *E. coli*, likely originating from a perinephric abscess. This emphasizes the importance of considering non-pulmonary sources of infection in patients with empyema. To our knowledge, there has been one prior published case report of a patient developing empyema after removal of chest tube 1 month prior following pneumonia [15]. Our patient’s course was complicated by chest tube dislodgement and formation of pleurocutaneous fistula requiring surgical decortication. While EN is increasingly rare since the advent of antibiotic therapy, our paper highlights the importance of early recognition and timely intervention in preventing serious complications, such as sepsis, respiratory failure, and bronchopleural fistula formation.
